# Supplementary material for: Informed baseline subtraction of proteomic mass spectrometry data aided by a novel sliding window algorithm
Source: Proteome Sci. 2016 Dec 7;14:19. doi: 10.1186/s12953-016-0107-8 (PMC5142289; doi:10.1186/s12953-016-0107-8)
Supplement: Additional file 1 — Morphological image analysis. An overview of the mathematical machinery used to define the top-hat operator. A small example is provided to demonstrate how the top-hat operator is applied to data. (PDF 240 kb) [file 12953_2016_107_MOESM1_ESM.pdf]

Morphological image analysis

The core concepts in mathematical morphology required to apply the top-hat operator are presented below. The concepts and definitions provided here assume some familiarity with the notational conventions regarding compositions of functions. Most university level calculus textbooks cover compositions of functions for further information, e.g., Anton et al. (2012). The definitions of a morphological *structuring element*, *dilation*, *erosion*, *opening* and *top-hat* presented below can also be found in Dougherty (1992); Soille (1999); Gil and Kimmel (2002); Van Droogenbroeck and Buckley (2005).

Definitions and concepts

A structuring element (SE) is a small set that acts on given data or images. For linear TOF-MS data, a SE is simply a one-dimensional line-segment, or window, passed over the vector of spectral intensities. In the context of morphological image analysis, the SEs used are centred (the median SE value is 0), symmetric (the SE behaves the same either side of the centre) and flat (SE is or the same dimension as the data). Non-flat SEs are not ideal for the current application as they require a known function or weightings to be applied within the sliding window.

Definition 1 *For the sets $X \subset \mathbb{Z}^p$ and $B \subset \mathbb{Z}^p$, $p \in \mathbb{Z}^+$, and the function f defined over X, the erosion of X by B is defined as,*

$$\begin{aligned} \epsilon_B(f)(x) &:= (f \ominus B)(x) \\ &:= \inf_{b \in B} f(x+b), \end{aligned}$$

for each element x in X. The dilation is similarly defined,

$$\begin{aligned} \delta_B(f)(x) &:= (f \oplus B)(x) \\ &:= \sup_{b \in B} f(x+b). \end{aligned}$$

Erosions and dilations can be thought of as rolling minimums and maximums, respectively, over the spectral values. Sometimes the sets X and B in Defintion 1 are defined over $\mathbb{R}^p$ Dougherty (1992); Van Droogenbroeck and Buckley (2005) but this is rarely implemented for data other than $X \subset \mathbb{Z}^p$ in practice.

Definition 2 *The application of a morphological erosion followed by a morphological dilation to a set X is the morphological opening,*

$$\begin{aligned} \omega_B(f)(x) &:= \delta_B(\epsilon_B(f))(x) \\ &:= ((f \ominus B) \oplus B)(x). \end{aligned}$$

In the context of linear TOF-MS data, a morphological opening is a non-linear estimation of background signal of the one-dimensional spectrum on X. The opening has the desirable property that it is never returns values greater than the observed signal, i.e. $\omega_B \leq f \ \forall \ x \in X$.

Definition 3 *The top-hat operator is defined as the removal of the opening from the original signal f,*

$$\tau_B(f)(x) := f(x) - \omega_B(f)(x).$$

The result of applying the top-hat operator to proteomic TOF-MS is the estimation of the true signal by removing the estimated background signal from f on X. Because of the $\omega_B(f) \leq f\ (\forall\ x)$ property of morphological openings, the top-hat operator provides a background estimate and removal without risk of creating negative signal, since it is a physical impossibility of the system. Such properties cannot be guaranteed by local regression of local minima.

Example of the top-hat operator

To illustrate the morphological operators that have been defined, we consider a simple example. Let $f = \{a_x\}_{x=1}^{13}$ be a series and define a flat SE, $B = \{b_j\}_{j=1}^{5} = \{-2, -1, 0, 1, 2\}$ with

$$f(x) = \begin{cases} a_1 & \text{if } x < 1 \\ a_x & \text{if } x = 1, 2, \ldots, 13 \\ a_{13} & \text{if } x > 13, \end{cases}$$

where

$$\{a_x\} = \{\ 6\quad 11\quad 12\quad 14\quad 7\quad 10\quad 13\quad 9\quad 12\quad 15\quad 8\quad 11\quad 10\ \}.$$

The erosion at $x = 4$ is calculated,

$$\begin{aligned} \epsilon_B(f)(4) &= \inf_{b \in B} f(4 + b) \\ &= \inf\{11, 12, 14, 7, 10\} = 7. \end{aligned}$$

Given the erosions for $x = 2, 3, 4, 5, 6$ are $6, 6, 7, 7, 7$, respectively, the morphological opening at $x = 4$ is

$$\begin{aligned} \omega_B(f)(4) &= \delta_B(\epsilon_B(f))(4) \\ &= \sup_{b' \in B} \{\epsilon_B(f)(4 + b')\} \\ &= \sup\left\{\inf_{b \in B} f(2 + b), \inf_{b \in B} f(3 + b), \inf_{b \in B} f(4 + b), \inf_{b \in B} f(5 + b), \inf_{b \in B} f(6 + b)\right\} \\ &= \sup\{6, 6, 7, 7, 7\} = 7. \end{aligned}$$

Therefore, the top-hat operator result for $x = 4$ is

$$\tau_B(f)(4) = f(4) - \omega_B(f)(4) = 14 - 7 = 7.$$

The operations with ϵ_B, ω_B and τ_B using the flat SE, $B = \{-2, -1, 0, 1, 2\}$, on the entire signal $f(x)$ can be observed in Figure 1.

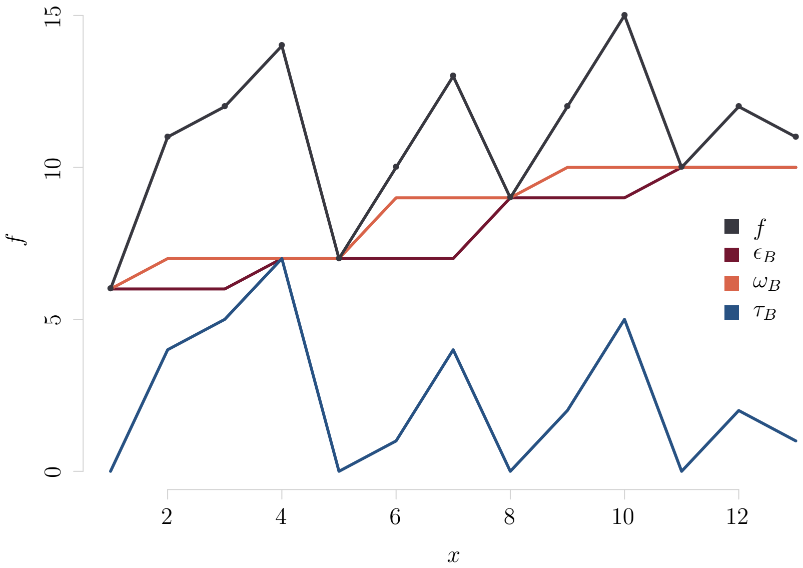

Figure 1: Baseline subtraction on an example spectrum using the top-hat operator (see main text for details): an demonstration of the erosion, opening and top-hat operators (ϵ_B, ω_B and τ_B, respectively) on a set f.

References

H Anton, I Bivens, and S Davis. *Calculus: early transcendentals.* John Wiley, 10th edition, 2012.

E Dougherty. *Mathematical Morphology in Image Processing.* Marcel-Dekker, New York, 1992.

J Y Gil and R Kimmel. Efficient dilation, erosion, opening, and closing algorithms. *IEEE Transactions on Pattern Analysis and Machine Intelligence*, 24(12):1606–1617, 2002.

P Soille. *Morphological Image Analysis: Principles and Applications.* Springer-Verlag New York, Inc., Secaucus, NJ, USA, 1999.

M Van Droogenbroeck and M J Buckley. Morphological erosions and openings: fast algorithms based on anchors. *Journal of Mathematical Imaging and Vision*, 22(2-3):121–142, 2005.
