# Supplementary material for: Informed baseline subtraction of proteomic mass spectrometry data aided by a novel sliding window algorithm
Source: Proteome Sci. 2016 Dec 7;14:19. doi: 10.1186/s12953-016-0107-8 (PMC5142289; doi:10.1186/s12953-016-0107-8)
Supplement: Additional file 2 — Six transformation functions on each of the six datasets. The effect of the six transformation functions, t 0- t 5, on a spectrum from each of the six datasets. (PDF 4669 kb) [file 12953_2016_107_MOESM2_ESM.pdf]

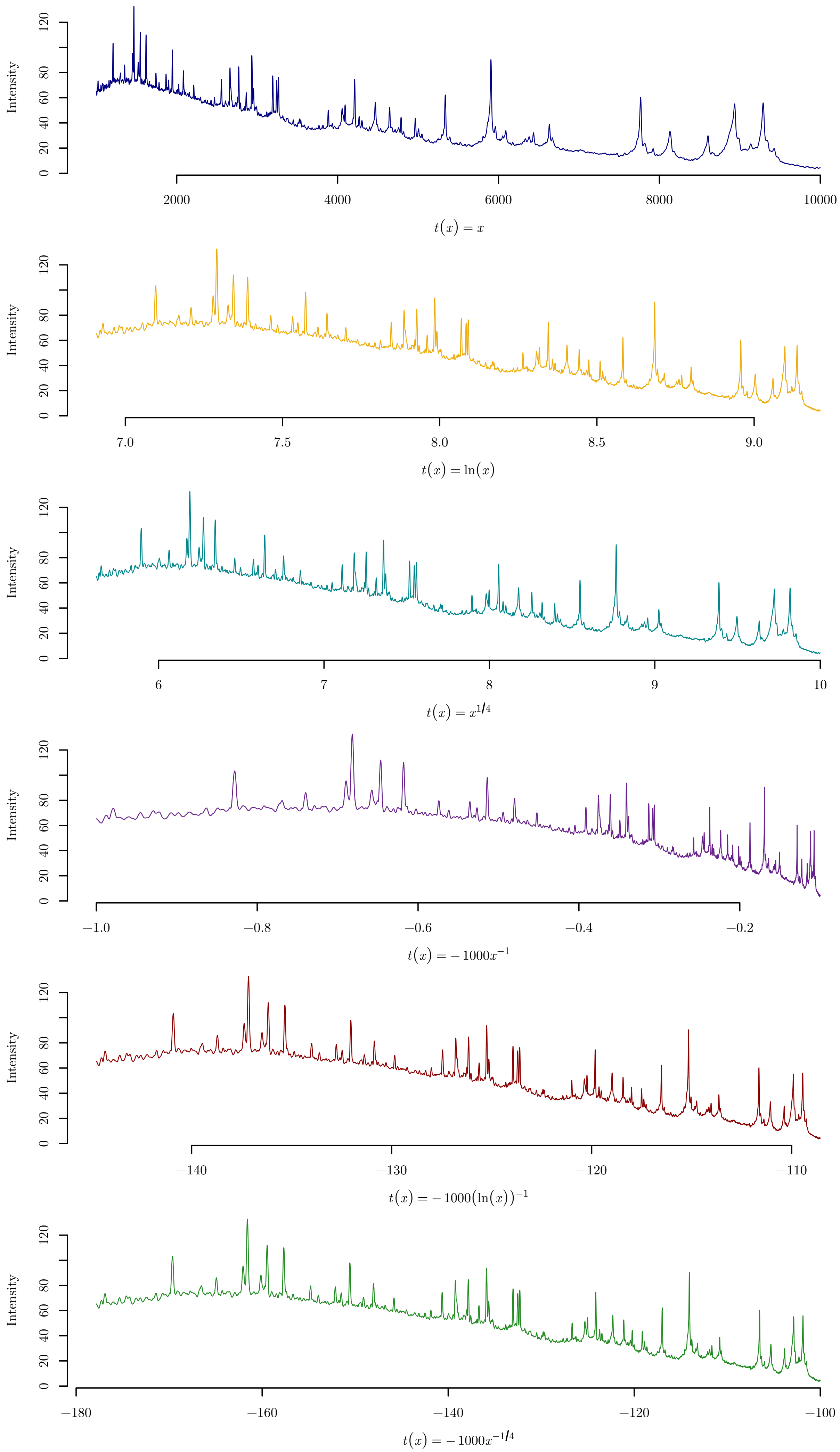

Figure 1: The effect of the six transformation functions, t_0-t_5, on a spectrum from the Fiedler dataset.

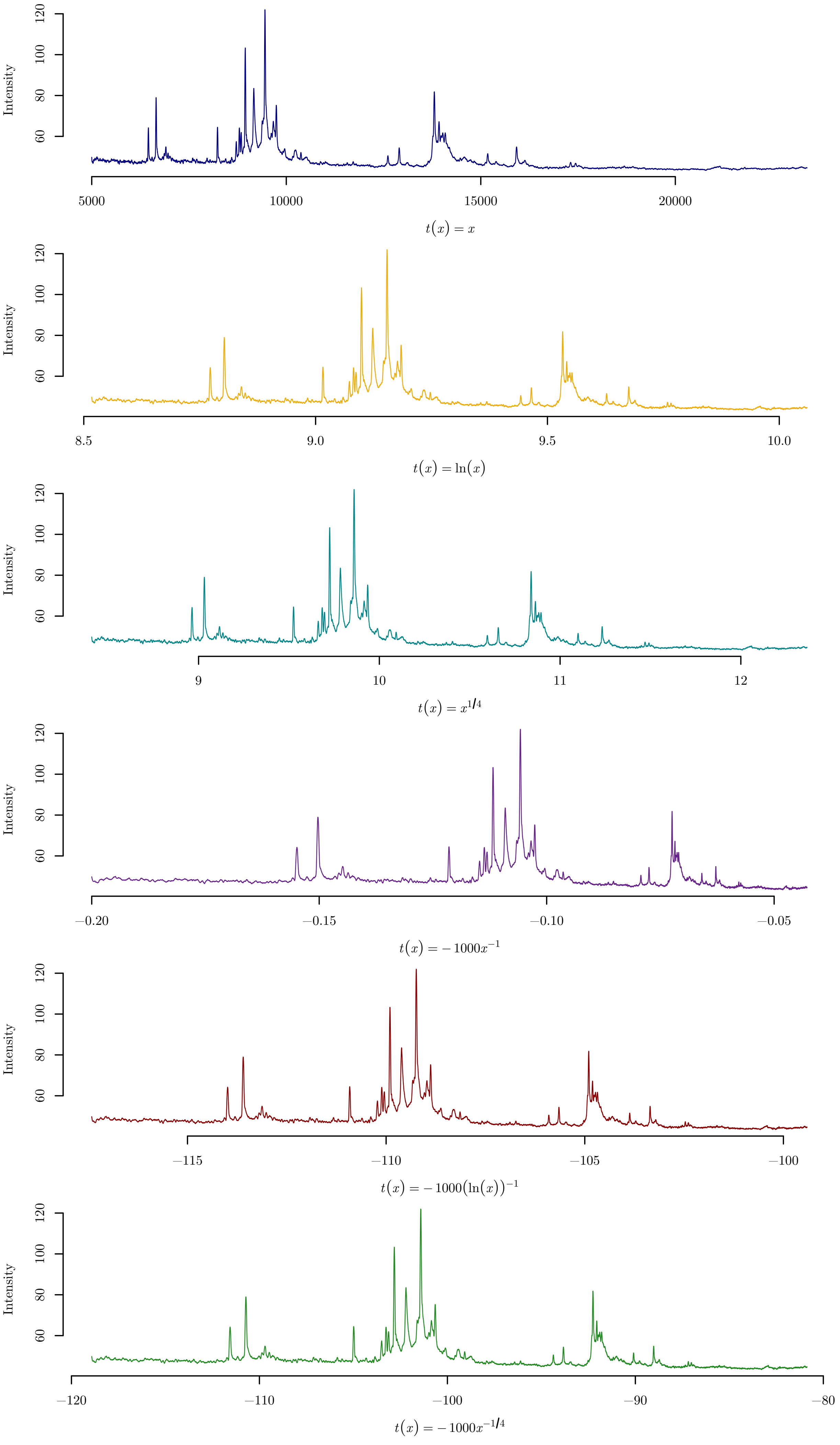

Figure 2: The effect of the six transformation functions, t_0-t_5, on a spectrum from the Yildiz dataset.

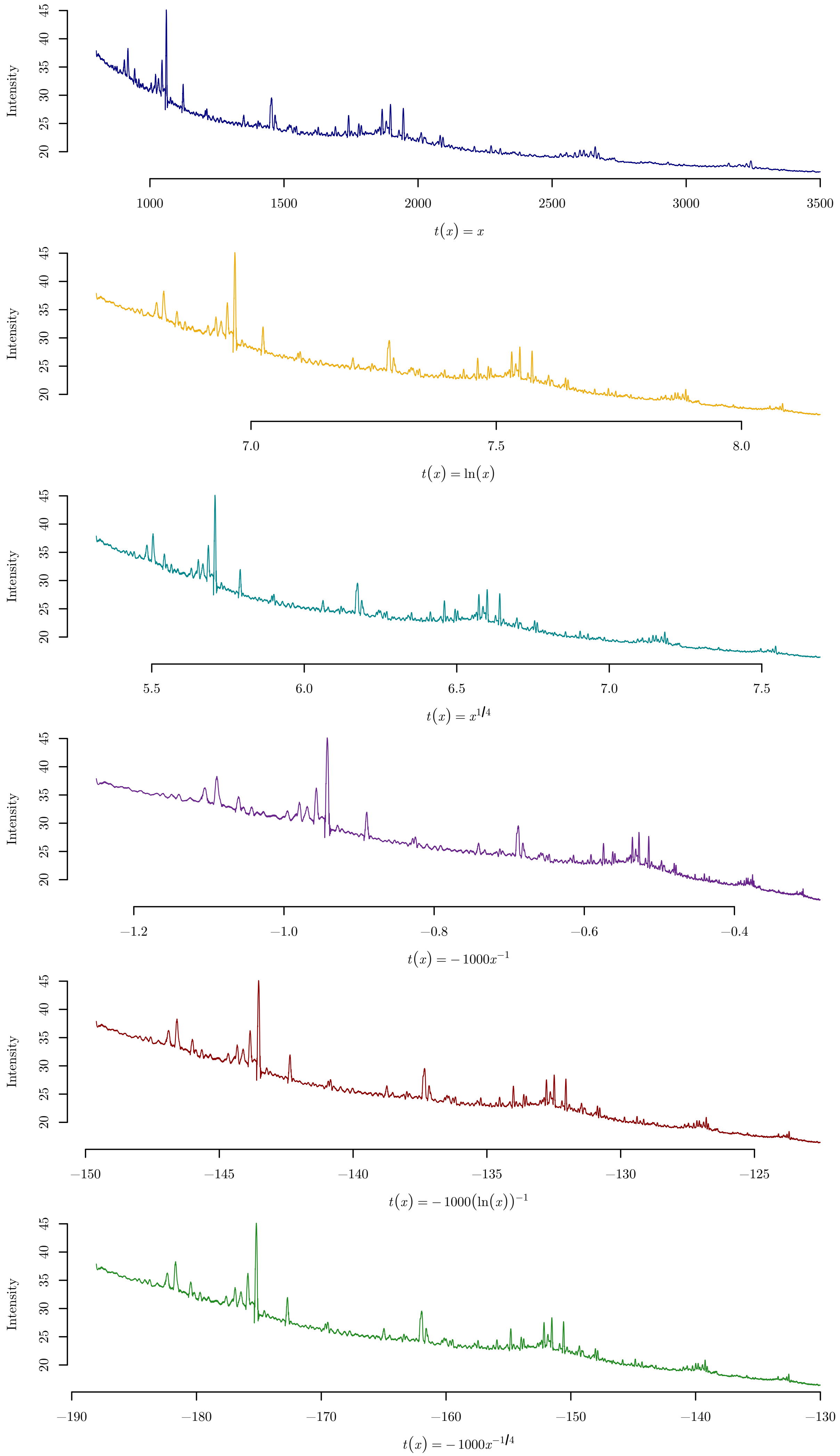

Figure 3: The effect of the six transformation functions, t_0-t_5, on a spectrum from the Wu dataset.

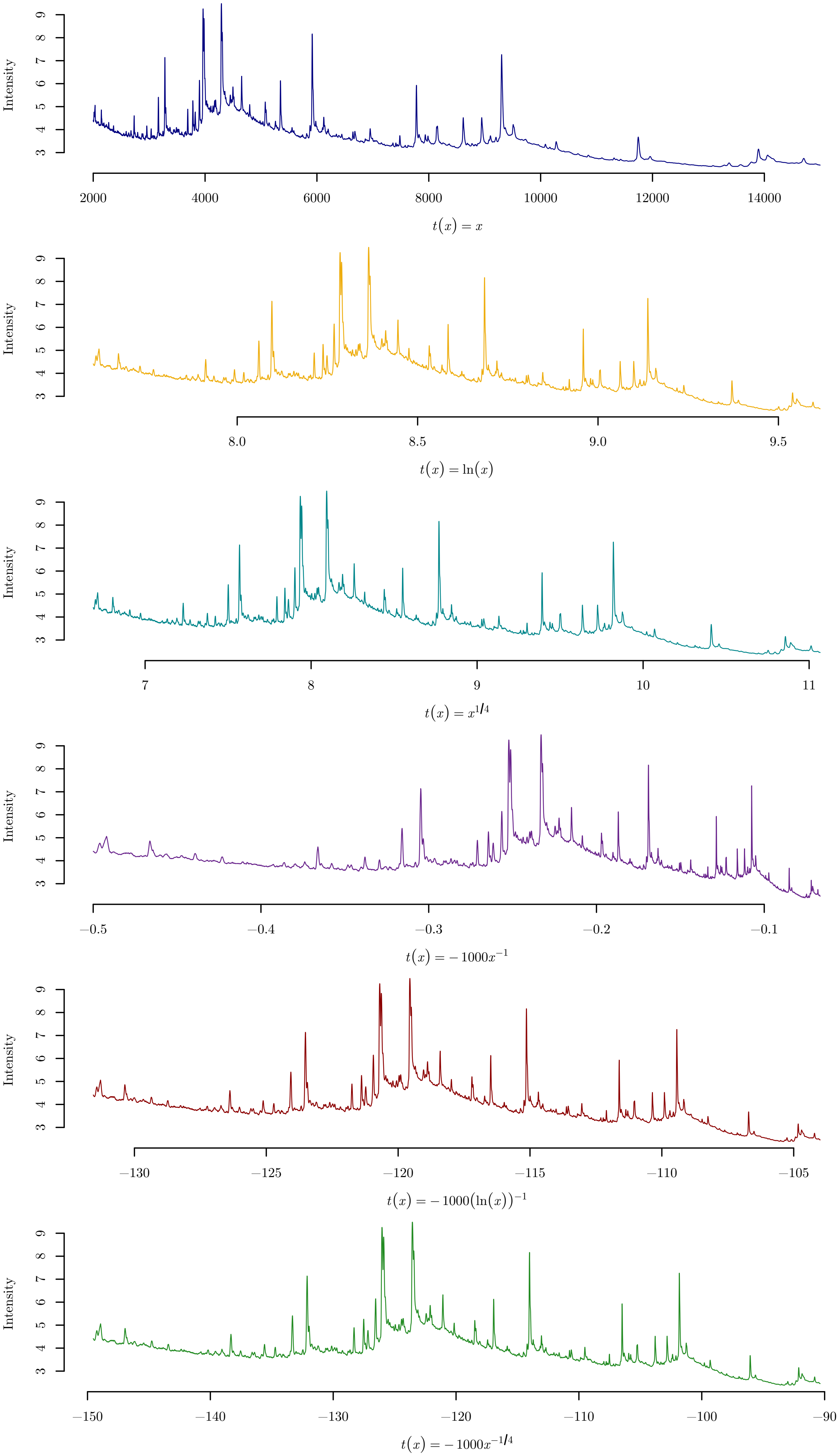

Figure 4: The effect of the six transformation functions, t_0-t_5, on a spectrum from the Adam dataset.

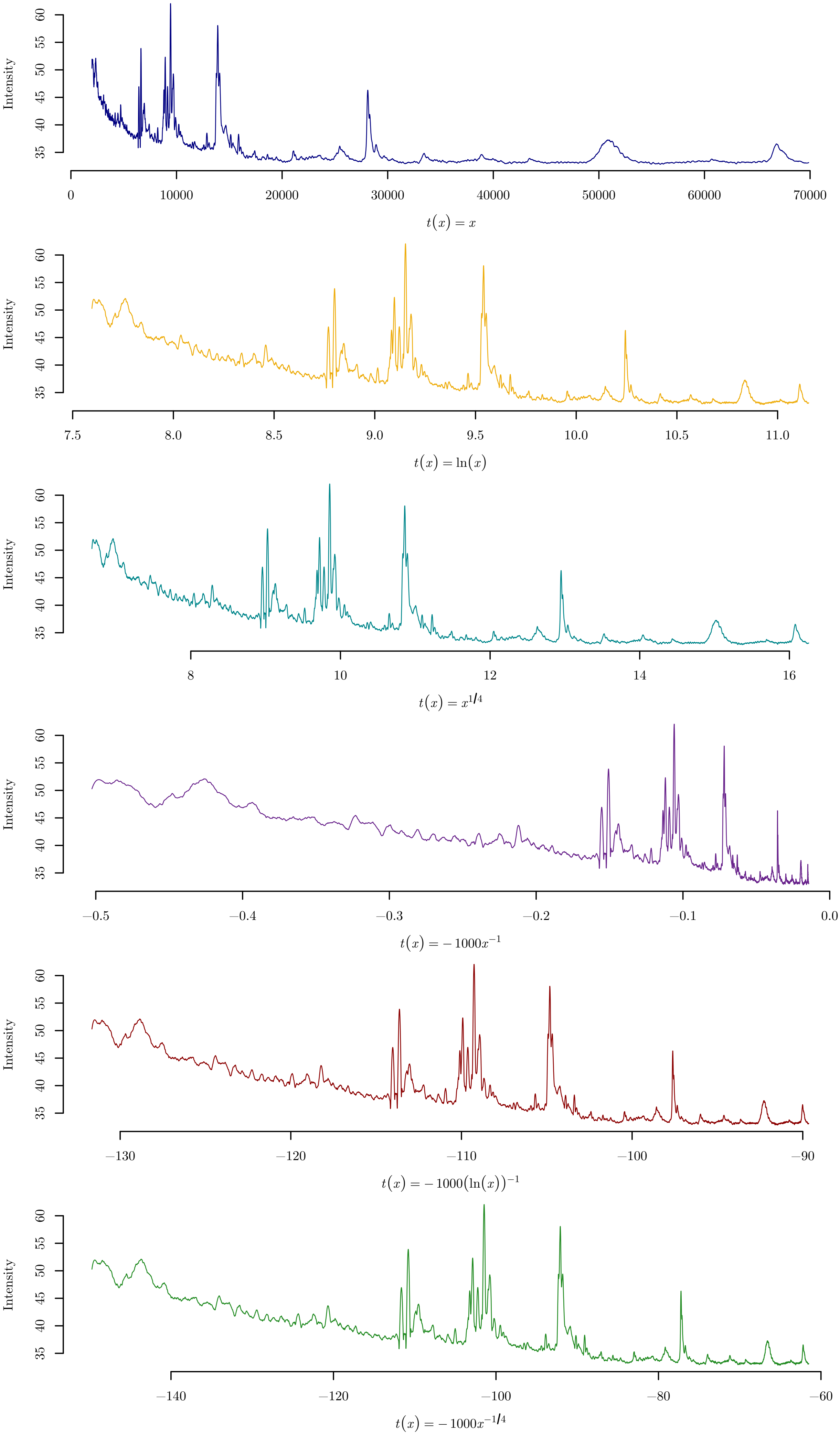

Figure 5: The effect of the six transformation functions, t_0-t_5, on a spectrum from the Taguchi dataset.

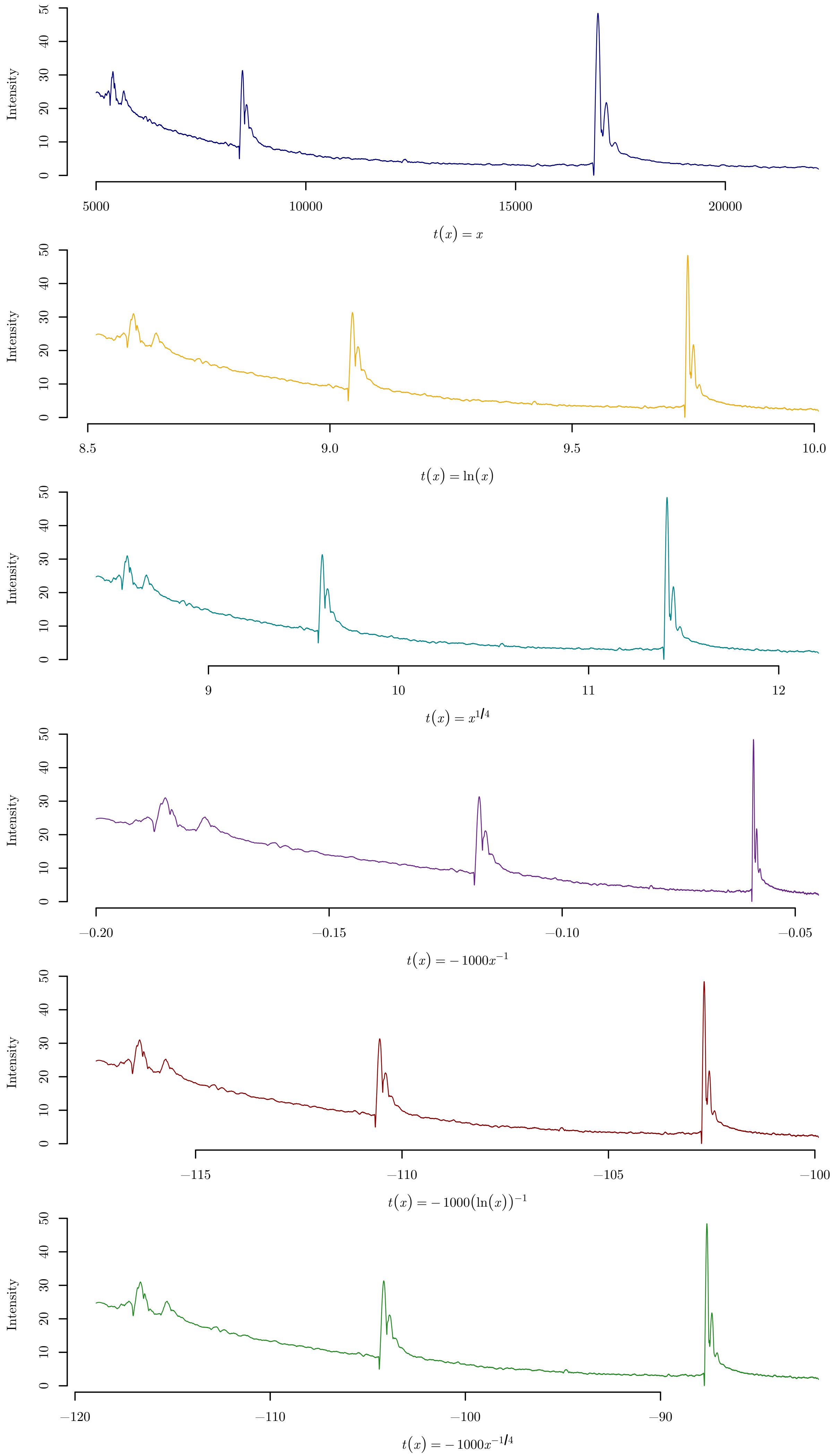

Figure 6: The effect of the six transformation functions, t_0-t_5, on a spectrum from the Mantini dataset.
