# Supplementary material for: Informed baseline subtraction of proteomic mass spectrometry data aided by a novel sliding window algorithm
Source: Proteome Sci. 2016 Dec 7;14:19. doi: 10.1186/s12953-016-0107-8 (PMC5142289; doi:10.1186/s12953-016-0107-8)
Supplement: Additional file 3 — EPCP and m/z-axis transformation optimisation. The AMASE results on each of the six datasets for varying EPCP and m/z-axis transformations as well as an illustrative plot of estimated peak widths on the t 2(m/z) scale. (PDF 633 kb) [file 12953_2016_107_MOESM3_ESM.pdf]

Estimated peak coverage proportion and m/z-axis transformation optimisation

Figure 1 and Table 1 present the AMASE (average mean absolute scaled error) values for the six datasets. For each dataset, Figure 1 displays a grid of the input arguments which are the m/z-axis transformation and the EPCP (estimated peak coverage proportion). The colour of the blocks at the intersection of these combinations depicts the AMASE value obtained. Darker blocks indicate smaller, and thus preferred, AMASE values. Table 1 is simply a tabular presentation of the results shown in Figure 1.

It is worth noting the spectra with fewer peaks generally had larger AMASE values; this is a function of the standardising constant of the MASE,

$$\frac{1}{\frac{1}{n-1}\sum_{i=2}^{n}|\tau_i^* - \tau_{i-1}^*|}.$$

Fewer peaks will generally imply less absolute change in adjacent signals and thus a larger standardising constant.

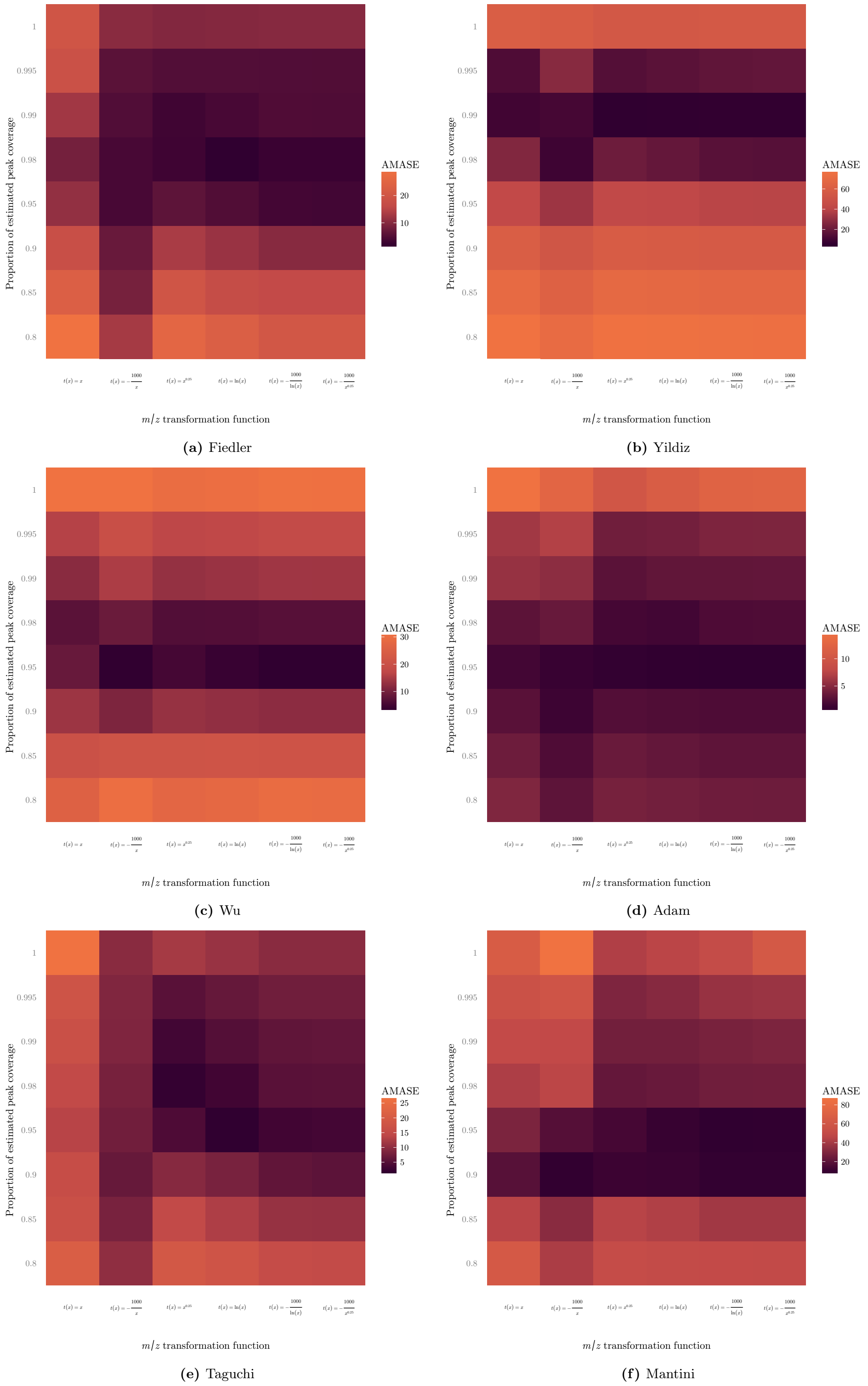

Figure 1: Average mean absolute scaled error (AMASE) heatmaps: AMASE values for each of the six datasets under different combinations of m/z transformation and estimated peak coverage proportion (EPCP).

Table 1: Average mean absolute scaled error (AMASE) when using structuring element (SE) sizes corresponding to different estimated peak coverage proportions (EPCPs) for each of the selected shortlisted transformations on each of the six datasets.

EPCP	$t_0(x) = x$	$t_1(x) = \frac{-1000}{x}$	$t_2(x) = x^{1/4}$	$t_3(x) = \ln x$	$t_4(x) = \frac{-1000}{\ln x}$	$t_5(x) = \frac{-1000}{x^{1/4}}$
			Fiedler			
1	19.8	10.1	9.5	9.7	9.9	9.9
0.995	18.4	5.5	4.8	4.7	4.7	4.6
0.99	12.4	4.7	3.0	3.7	4.5	4.5
0.98	8.2	3.7	2.8	1.3	2.3	2.3
0.95	11.0	3.7	5.8	4.6	3.3	3.1
0.9	17.8	6.9	13.2	11.7	9.9	10.0
0.85	23.2	8.3	19.8	16.9	16.1	16.2
0.8	29.4	12.9	25.5	22.9	20.2	20.2
			Yildiz			
1	59.7	58.3	54.9	54.6	55.6	55.7
0.995	12.9	27.4	14.3	15.8	17.5	18.0
0.99	9.3	10.7	5.0	5.2	5.5	5.5
0.98	25.7	8.6	20.9	18.4	15.4	14.9
0.95	42.3	32.6	41.6	41.0	39.7	39.5
0.9	59.7	52.7	58.4	57.7	56.7	56.6
0.85	70.4	62.9	69.2	68.1	67.2	67.0
0.8	77.1	71.5	76.6	75.9	75.0	74.9
			Wu			
1	30.9	30.8	29.2	29.7	30.4	30.2
0.995	16.1	19.5	16.9	17.2	18.0	17.9
0.99	12.0	15.2	13.0	13.5	14.1	14.0
0.98	7.4	9.0	6.7	6.7	7.2	7.1
0.95	8.8	3.4	5.4	4.1	3.3	3.3
0.9	13.8	10.9	13.3	12.8	12.3	12.3
0.85	20.1	20.9	20.9	21.1	20.7	20.7
0.8	25.4	29.7	27.0	27.7	28.8	28.7
			Adam			
1	14.8	12.7	10.1	11.2	12.3	12.4
0.995	6.2	7.1	3.8	3.9	4.5	4.5
0.99	5.7	5.2	2.7	3.0	3.1	3.1
0.98	2.8	3.4	1.6	1.5	2.1	2.1
0.95	1.5	1.0	0.7	0.6	0.6	0.5
0.9	2.6	1.2	2.3	2.2	2.1	2.1
0.85	3.7	2.2	3.4	3.2	2.9	2.9
0.8	4.6	2.9	4.1	3.9	3.7	3.7
			Taguchi			
1	26.5	9.6	12.0	11.0	9.6	9.7
0.995	17.7	8.9	5.5	6.5	7.5	7.5
0.99	16.4	8.8	3.5	5.1	6.1	6.2
0.98	14.8	8.1	2.2	3.3	5.5	5.6
0.95	13.6	7.6	4.5	1.8	3.3	3.6
0.9	15.7	6.7	9.4	8.2	6.2	5.8
0.85	16.5	8.2	14.7	12.7	10.8	10.7
0.8	20.7	10.2	18.9	17.7	15.4	14.9
			Mantini			
1	66.1	86.4	42.8	45.6	50.6	63.0
0.995	55.1	58.2	29.6	31.8	36.3	37.2
0.99	49.3	48.3	26.2	26.2	27.9	28.9
0.98	42.1	45.9	22.3	23.4	25.8	25.9
0.95	28.8	18.1	13.9	9.5	7.6	7.9
0.9	18.8	8.1	11.0	10.6	8.5	8.5
0.85	45.3	32.4	45.0	42.9	38.9	38.9
0.8	63.1	41.6	51.2	49.7	49.2	49.0

Illustrative example of estimated peak coverage proportion on the $t_2(m/z)$ scale for the Fiedler dataset

Figure 2 represents the estimated peak widths for the 16 spectra in the Fiedler dataset on the $t_2\,(m/z)$ scale, where peak regions are found using a repeated lower convex hull algorithm presented in the main article.

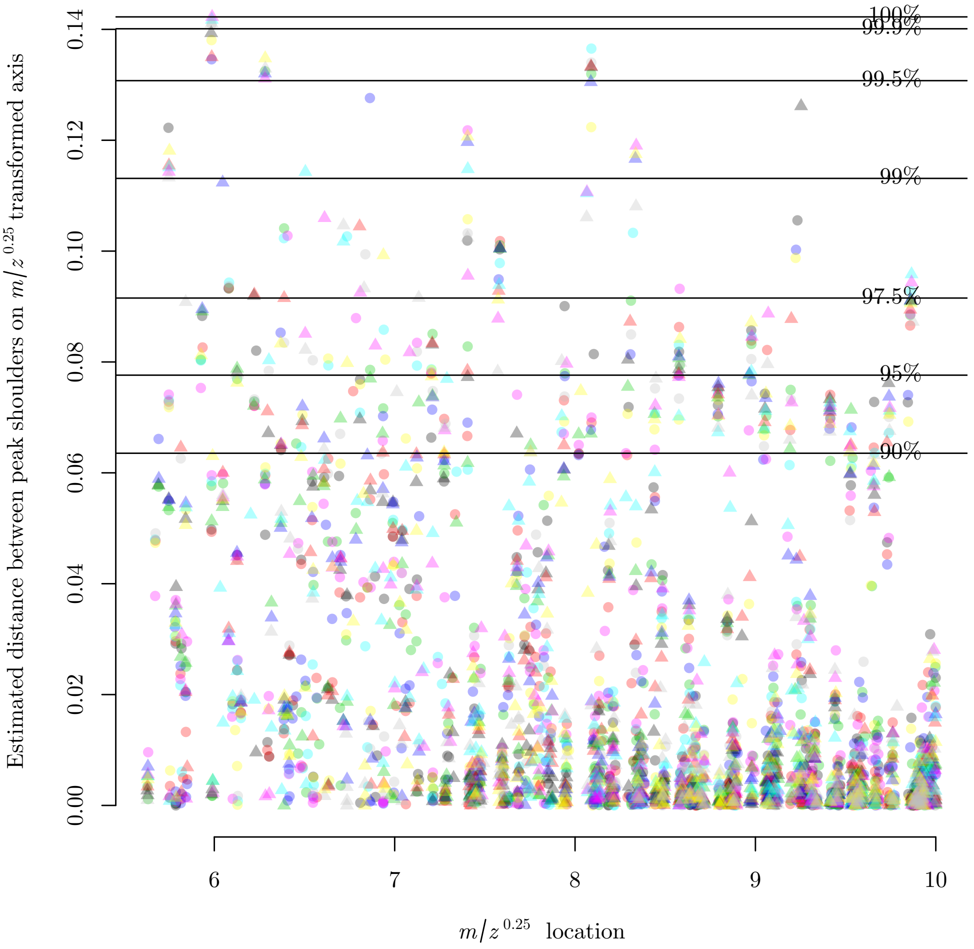

Figure 2: Estimated peak widths on the $t_2(m/z)$ scale. A scatter plot of peak location versus peak widths on the quartic root scale in the Fiedler data as determined by the peak width estimation algorithm given in Figure 6 (primary paper). The colour and shape combination of each point corresponds to the 16 spectra in the data. It is anticipated that the m/z transformation removes the relationship between peak location and peak width, as demonstrated. The horizontal lines denote the proportion of peak widths that lie below it. By choosing a SE size that corresponds with the peak width at the 99.5% horizontal line, for example, the top-hat operator is only at risk of 'undercutting' 0.5% of peaks that are wider than the SE width.
