# Supplementary material for: Informed baseline subtraction of proteomic mass spectrometry data aided by a novel sliding window algorithm
Source: Proteome Sci. 2016 Dec 7;14:19. doi: 10.1186/s12953-016-0107-8 (PMC5142289; doi:10.1186/s12953-016-0107-8)
Supplement: Additional file 4 — CLSA examples. Examples with calculations to demonstrate the continuous line segment algorithm. (PDF 308 kb) [file 12953_2016_107_MOESM4_ESM.pdf]

Examples of the CLSA on continuous data

To illustrate how the CLSA works, consider two cases of the algorithm in returning the erosion in Figures 1 and 2.

Figure 1 shows a case where $\epsilon_B(f)(x_{12}) = 3$ using a SE of size $k = 3$. It can be seen that,

$$\theta_{w^{\triangledown}_{12}-1} = \theta_{10} = 3 \neq \theta_{w^{\vartriangle}_{12}} = \theta_{13} = 4,$$

but,

$$\theta_{w^{\triangledown}_{12}} = \theta_{11} = 3 = \theta_{w^{\vartriangle}_{12}+1} = \theta_{14}.$$

Therfore the desired result is also achieved using the CLSA as,

$$r_{\min}\left(f\left(x_{12}\right)\right) = g(x_{w^{\vartriangle}_{12}}) = g(x_{13}) = 3 = \epsilon_B(f)(x_{12}).$$

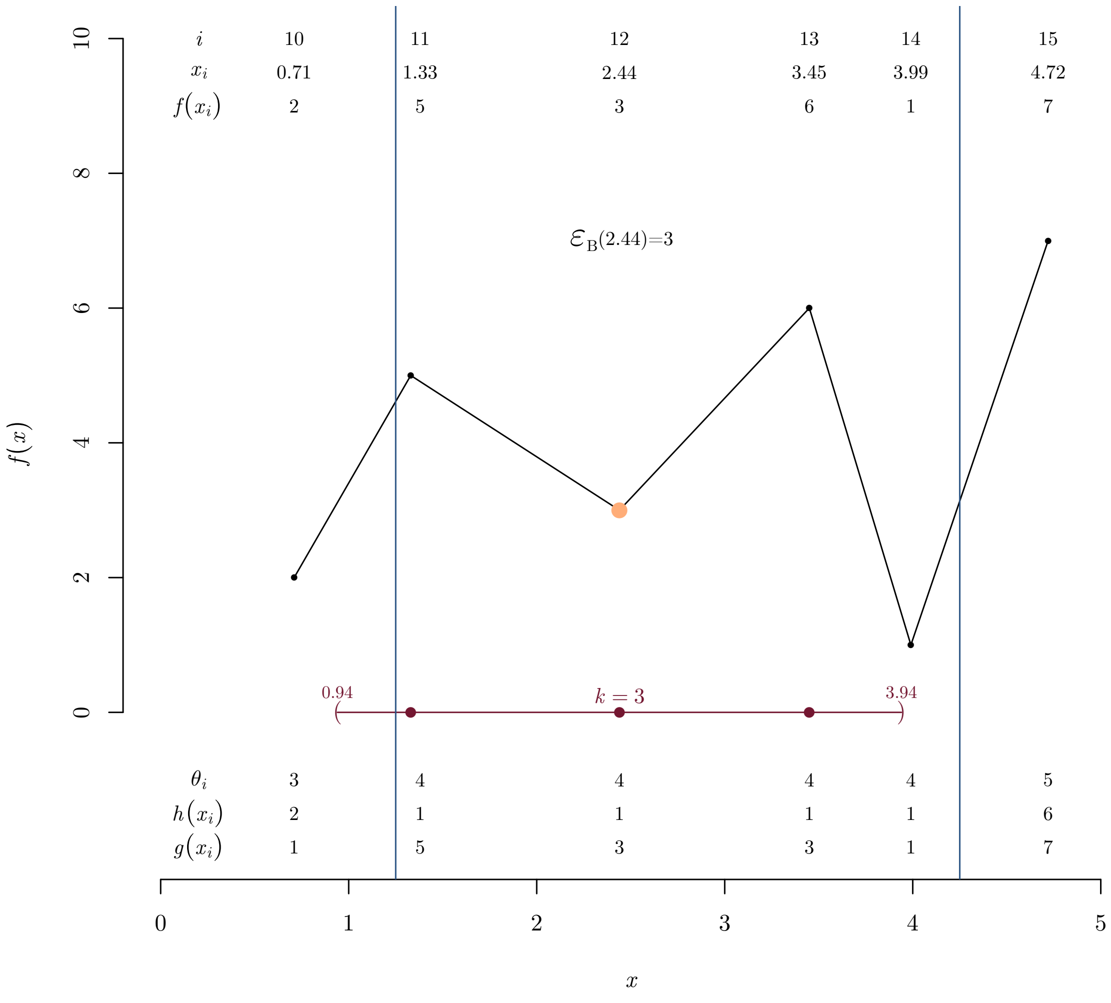

Figure 1: An example of data for $x_i = x_{12} = 2.44$ and $k = 3$ where $\theta_{w^{\triangledown}_i} = \theta_{w^{\vartriangle}_i+1}$ (i.e. $\theta_{w^{\triangledown}_{12}} = \theta_{w^{\vartriangle}_{12}+1} = 4$) and the computation required to return the result of the CLSA (the tan coloured point $f(2.44) = 3$).

Figure 2 is a different case in the CLSA where $\theta_{w_i^{\triangledown}-1} = \theta_{w_i^{\vartriangle}}$, as opposed to the case shown in Figure 1 where $\theta_{w_i^{\triangledown}} = \theta_{w_i^{\vartriangle}+1}$. To obtain the erosion of point $x_i = x_9 = 2.44$ for $k = 3$ using the CLSA, observe that

$$\theta_{w_9^{\triangledown}} = \theta_8 = 3 \neq \theta_{w_9^{\vartriangle}+1} = \theta_{11} = 4,$$

and

$$\theta_{w_9^{\triangledown}-1} = \theta_7 = 3 = \theta_{w_9^{\vartriangle}} = \theta_{10}.$$

Therefore, the result of the CLSA erosion is

$$r_{\min}\left(f\left(x_9\right)\right) = h(x_{w_9^{\triangledown}}) = h(x_8) = 3.$$

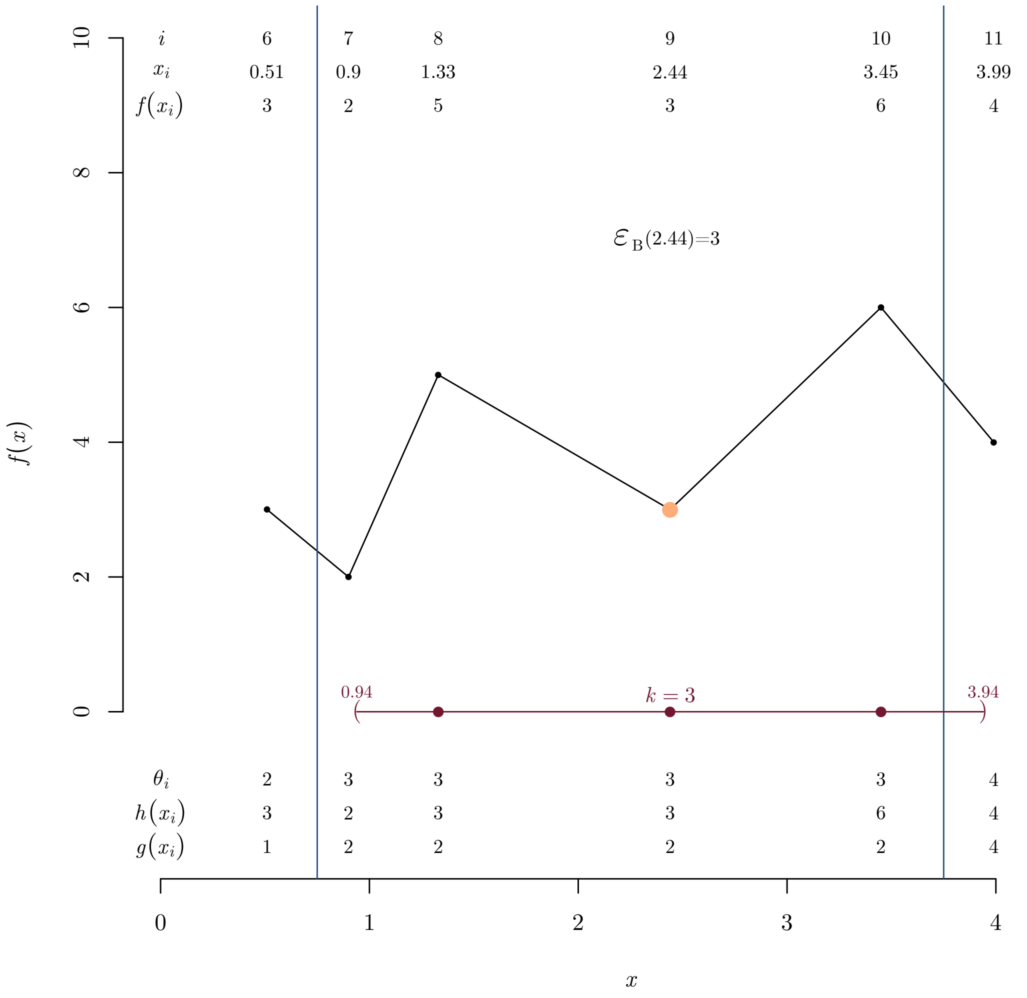

Figure 2: An example of data where $\theta_{w_i^{\triangledown}-1} = \theta_{w_i^{\vartriangle}}$ and the computation required for the continuous line segment algorithm.
