# Supplementary material for: Informed baseline subtraction of proteomic mass spectrometry data aided by a novel sliding window algorithm
Source: Proteome Sci. 2016 Dec 7;14:19. doi: 10.1186/s12953-016-0107-8 (PMC5142289; doi:10.1186/s12953-016-0107-8)
Supplement: Additional file 5 — CLSA and naive algorithm computational times for simulated data. R code to produce the top-hat baseline subtraction computational time results shown in Table 3. (PDF 30.9 kb) [file 12953_2016_107_MOESM5_ESM.pdf]

R-code to reproduce Table 3 of main paper

```
##########################################################################################
##########################################################################################
################################## preamble and set-up ##################################
##########################################################################################
##########################################################################################

# required to install github packages
# see http://cran.r-project.org/web/packages/devtools/README.html
library(devtools)

#install CLSA package
devtools::install_github('tystan/clsa')
library(clsa)
# documentation
?clsa_min

# latex formatted table output
library(xtable)

# function to create x values (m/z values)
get_x_coords<-function(n) return(sort(rbeta(n,1,3)))
# function to create intensity values
get_f_signal<-function(n) return(rchisq(n,10))


##########################################################################################
##########################################################################################
################################## testing comp times ###################################
##########################################################################################
##########################################################################################

# creating a data.frame with the following columns:
# * n: the number of m/z points
# * win: the window sizes
# * time_naiv: using the naive alg -- time taken for the row's 'n' and 'win' values
# * time_clsa: using the CLSA -- time taken for the row's 'n' and 'win' values

# ranges of dataset size and window size:
n_rng<-seq(1e4,1e5,by=1e4)
win_rng<-c(0.005,0.01,0.02,0.05,0.1,0.2)
# these times will be updated
time_naiv<-0
time_clsa<-0
# enumerate all n and win combinations
time_df<-as.data.frame(
  expand.grid(
     n=n_rng
    ,win=win_rng
    ,time_naiv=time_naiv
    ,time_clsa=time_clsa
  )
)
(n_df<-nrow(time_df)) # should be 6x10=60
time_df

# test each n and win combination 20 times,
# i.e., each dataset has 20 "spectra"
n_reps<-20
set.seed(12345) # make reproducible

# now iterate over rows of the data.frame for each "spectrum",
```

```
# and time the computation
for(j in 1:n_reps)
{
  for(i in 1:n_df)
  {

    # get n and win combination
    n<-time_df$n[i]
    this_win<-time_df$win[i]
    # update console of progress
    cat("::: Iteration",(j-1)*n_df+i,"of",n_df*n_reps,"::: ")
    cat("n =",n,"and window size = ",this_win,":::\n")
    # randomly generate x and f
    x<-get_x_coords(n)
    f<-get_f_signal(n)

    # time the computations, add to previous "spectra" times
    time_df$time_clsa[i]<-time_df$time_clsa[i]+
      system.time(a<-clsa_max(x,clsa_min(x,f,this_win),this_win))[3]
    time_df$time_naiv[i]<-time_df$time_naiv[i]+
      system.time(b<-naiv_max(x,naiv_min(x,f,this_win),this_win))[3]

    # if the results are not equal between the naiv and CLSA we have
    # a problem; ABORT!
    if(!all(a==b)) break;
  }
}
time_df

########################################################################################
################################### table of results ###################################
########################################################################################

# extract times for naiv and CLSA for printing
time_naiv<-data.frame(n=time_df$n,win=time_df$win,time=time_df$time_naiv,func="Naive")
time_clsa<-data.frame(n=time_df$n,win=time_df$win,time=time_df$time_clsa,func="CLSA")

# print the results!
xtable(
  cbind(
    xtabs(time ~ I(n/1e4) + win, data = time_naiv)
    ,NA
    ,xtabs(time ~ I(n/1e4) + win, data = time_clsa)
  )
  ,digits=1
)
```
